# The Effect of Different Additives on the Hydration and Gelation Properties of Composite Dental Gypsum

**DOI:** 10.3390/gels7030117

**Published:** 2021-08-11

**Authors:** Liang Ma, Qianting Xie, Amutenya Evelina, Wenjun Long, Cunfa Ma, Fengshan Zhou, Ruitao Cha

**Affiliations:** 1 Beijing Key Laboratory of Materials Utilization of Nonmetallic Minerals and Solid Wastes, National Laboratory of Mineral Materials, School of Materials Science and Technology, China University of Geosciences Beijing, Haidian District, Beijing 100083, China; Maxliang@cugb.edu.cn (L.M.); cugbxqt@163.com (Q.X.); 9103190001@cugb.edu.cn (A.E.); longwenjun@cugb.edu.cn (W.L.); 2103190084@cugb.edu.cn (C.M.); 2 CAS Key Laboratory for Biomedical Effects of Nanomaterials and Nanosafety, National Center for NanoScience and Technology, No. 11, Haidian District, Beijing 100190, China; chart@nanoctr.cn

**Keywords:** dental gypsum, linear expansion coefficient, 2 h flexural strength, stability, gelation properties

## Abstract

Dental mold gypsum materials require fine powder, appropriate liquidity, fast curing, and easy-to-perform clinical operations. They require low linear expansion coefficient and high strength, reflecting the master model and facilitating demolding. In this article, the suitable accelerators and reinforcing agents were selected as additives to modify dental gypsum. The main experimental methods used were to compare the trends of linear expansion coefficients of several commercially available dental gypsum products over 72 h and to observe the cross-sectional microstructure of cured bodies before and after dental gypsum modification using scanning electron microscopy. By adjusting the application of additives, the linear expansion coefficient of dental gypsum decreased from 0.26% to 0.06%, while the flexural strength increased from 6.7 MPa to 7.4 MPa at 2 h. Formulated samples showed good stability and gelation properties with linear expansion completed within 12 h. It is indicated that the performance of dental gypsum materials can be improved by adding additives and nanomaterials, which provided a good reference for clinical preparation of high-precision dental prosthesis.

## 1. Introduction

Models of oral tissues are used in dentistry to assess, treat, and manufacture indirect restorations [1]. As an essential auxiliary material, dental gypsum has been used for simulating oral cavity models. Put a certain amount of dental gypsum powder and water in a small rubber bowl to knead the gypsum slurry and then cast the slurry into the oral mold to prepare dental restoration, such as an inlay, a crown, a bridge, a partial denture, and a complete denture [2,3,4]. The manufacture of denture model is the basis of denture processing, and the dimensional stability of gypsum is fundamental to achieving a precise fit between dental structure and restorative material [5,6].

The main component of dental gypsum is α-hemihydrate gypsum dissolved and recrystallized in saturated steam medium or liquid water solution. Doctors usually choose the corresponding gypsum products to make prostheses by referring to various performance parameters of gypsum materials. For example, an ordinary dental stone should be used when making ordinary elastic dentures or movable dentures, and a dental stone with high strength and low expansion should be chosen when making precision prostheses such as fixed dentures and attachments [7,8]. According to the specification of the American Dental Association (ADA) [9], and the International Organization for Standardization (ISO 6873), dental gypsum can be classified into five types [10]:

Type I—Impression plaster. The linear expansion value is: ADA: 0–0.15%, ISO: 0–0.15%; Type II—Model plaster. The linear expansion value is: ADA: 0–0.30%, ISO: 0–0.30%; Type III—Dental stone. The linear expansion value is: ADA: 0–0.20%, ISO: 0–0.20%; Type IV—Dental stone (low expansion, high strength). The linear expansion value is: ADA: 0–0.10%, ISO: 0–0.15%; Type V—Dental stone (high expansion, high strength). The linear expansion value is: ADA: 0.10–0.30%, ISO: 0.16–0.30%.

After dental impression filling, the dental gypsum material begins to solidify, and the volume shrinkage of the gypsum material occurs at the early stage of solidification. When the mixture of a model material is rigid, the expansion in all directions will affect the size of the model [11,12]. On the one hand, the gypsum material will form calcium sulfate dihydrate crystal during hydration with continuous growth and re-formation of crystal, resulting in the expansion of gypsum volume; on the other hand, the dissolution of calcium sulfate dihydrate requires excessive water, which will form stomata after evaporation of water except for crystalline water, thereby significantly increasing the volume of gypsum [13,14,15].

Clinical studies show that the resin denture made of plaster model material with low expansion coefficient has the highest accuracy. To ensure the consistency between the models and the oral cavity conditions as well as improving patient satisfaction, dental gypsum requires an accuracy capable of reproducing details of an oral cavity, with excellent strength and high stability, which prevents the reproduced details from being broken or damaged [16,17]. Currently, modification methods of dental gypsum are divided into the following three types, according to additive addition ways.

The first way is to coat some inorganic and organic solutions on the surface of the gypsum model after solidification to solve problems of surface hardness and wear resistance of the gypsum model [18,19]. The second way is adding some salts, alkalis, and organic substances into water to form a new solution for mixing gypsum to help improve mechanical properties of the model during the mixing process of gypsum materials. However, the current application of this method is still limited, and the research hotspots focus on the application of disinfection and antimicrobial additives [20,21]. The third way employs the addition of some salt, alkali, or some organic substances grounded into a powder to the natural gypsum powder for improving the rheological and gelation properties of gypsum to produce a high-quality dental gypsum product [22,23]. This type of addition is currently the most widely used and has the most significant impact.

However, the research methods for the expansion properties of dental gypsum at home and abroad are still immature. In this study, the effects of additives on the linear expansion properties of dental gypsum were discussed by looking for effective solid additives and appropriate dosages. Since meeting the rheological properties and strength requirements of gypsum materials in clinical operations, the linear expansion coefficient is significantly reduced. It solves the problems of easy deformation, unstable size, and easy damage of dental gypsum materials, and its comprehensive performance is obviously better than existing dental gypsum products at home and abroad.

## 2. Results

In this study, single factor and orthogonal experiments were used to study the effects of various additives on the gelation properties of dental gypsum. The water–powder ratio was 23%, 0.1% BR was very beneficial to the adjustment of the weak-gel state, and the fixed dosage was 0.1%. Under the action of BR (borax cross-linking agent), the polymer network formed by the cross-linking of linear polymer additives enables the gypsum gelling system to effectively prevent the gypsum gel from sticking in the blending stage when it is regulated to the gel state; it also has a certain swelling-reducing effect, which can greatly improve the comprehensive blending properties and mechanical properties of dental gypsum.

### 2.1. Effect of Accelerant on the Gelation Characteristics of Dental Gypsum

Commonly used accelerants were acids (HCl, HNO_3_, H_2_SO_4_) and their salts. A systematic study on the accelerating effect of various anions and calcium indicated that K^+^ and ${\mathrm{SO}}_{4}^{2+}$ constituted the best accelerating anion-calcium pair. The effects of calcium sulfate dihydrate, potassium sulfate, and aluminum stearate on the gelation properties of dental gypsum were studied with 23% water powder ratio, 0.30% CPS (Copolymer water reducer), 0.03% SG (Sodium gluconate retarder), 0.10% BR (Borax cross-linker agent), and 2.00% Nano-SiO_2_ as experimental control group.

### 2.2. Linear Expansion Coefficient

**Figure 1:**
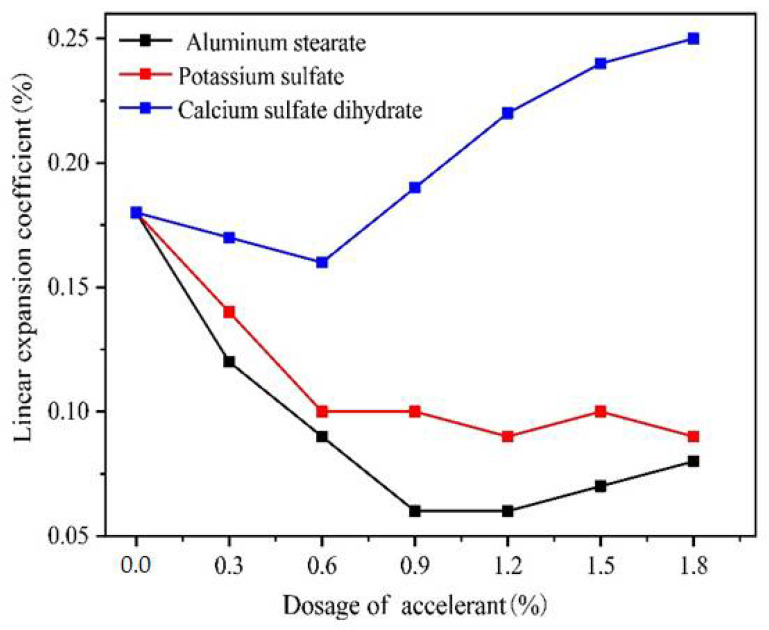
The effect of accelerant on the linear expansion coefficient of dental gypsum at different dosages (ww^−1^).

Three kinds of accelerants were selected in this experiment, and their effects on linear expansion coefficient of dental gypsum were shown in Figure 1.

When the dosage of calcium sulfate dihydrate exceeded 0.60%, the linear expansion value increased significantly. A small amount of potassium sulfate and aluminum stearate could significantly reduce the linear expansion coefficient of dental gypsum, among which the inhibitory effect on linear expansion of aluminum stearate was most obvious. When the dosage of aluminum stearate reached 0.90%, the 2 h linear expansion coefficient of dental gypsum was as low as 0.06%, which obviously weakened the formation of setting expansion. As the dosage of potassium sulfate and aluminum stearate continued to increase, the linear expansion coefficient showed a trend of slowly rising or basically unchanged.

When the crystal of hemihydrate gypsum was covered with an adsorbed layer of water molecules, the accelerant stabilized the layer and arranged the neighboring water molecules, resulting in a multilayer water structure. As the area and depth of coverage of the adsorbed water layer increased, the dissolution rate increased, thereby accelerating the reaction rate and shortening the setting time [24]. Given the influence of aluminum stearate on the setting time, when the final setting time was 6–12 min, the adjustable range of aluminum stearate was 0.30–1.20%.

**Figure 2:**
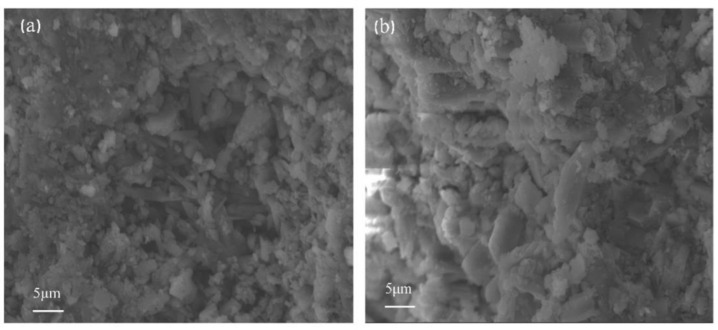
SEM images of gypsum (multiple = 3.5 k, scale = 5.00 μm) (**a**) blank dental gypsum, (**b**) 0.80% aluminum stearate.

Figure 2a,b shows cross-sectional morphologies of a blank dental gypsum sample and dental gypsum sample with 0.80% aluminum stearate observed under the electron scanning microscope, respectively. The water–powder ratio was 27% of standard consistency water demand. Compared with the blank group, after adding aluminum stearate, calcium sulfate dihydrate showed a larger crystal size, fewer inter-crystalline bonds, and a looser structure. According to the theory of expansion energy (crystal with unit mass, the expansion energy of crystal with coarse crystal is smaller), the 2 h linear expansion coefficient of dental gypsum was significantly reduced, which was consistent with the experimental results.

### 2.3. Mechanical Properties

**Figure 3:**
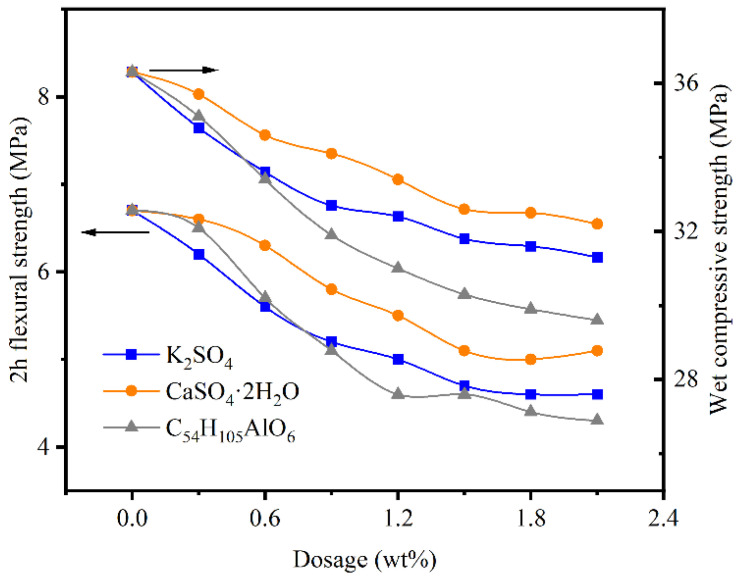
The effect of accelerant on the mechanical strength of dental gypsum.

Figure 3 shows that the effect of the accelerant on mechanical properties was mainly reflected in the 2 h flexural strength. After adding 0.9% aluminum stearate and potassium sulfate, 2 h flexural strength loss was 23.90% and 22.40%, respectively, and wet compressive strength loss was 9.90% and 12.10%, respectively. The results indicated that potassium sulfate was suitable for a shorter setting time, and aluminum stearate was suitable for a small amount of use to prevent a sharp decrease in strength.

It is worth noting that, when the dosage of potassium sulfate is too large, on the surface of the gypsum model appears a blooming phenomenon, which would affect the appearance of the model and destroy the compactness of the internal structure, leading to a sharp decrease in the strength and durability of the gypsum.

### 2.4. Effect of the Retarder on Linear Expansion Coefficient

In the application process of dental gypsum, a retarder was used as one of the necessary admixtures to adjust the setting time and linear expansion coefficient of the gypsum slurry to meet the operating requirements. At present, the commonly used gypsum retarders could be roughly divided into three categories: inorganic salts, organic acids, and organic macromolecules.

The effects of JR (methionine retarder), SG (sodium gluconate retarder) and GR (bone glue retarder) on the expansion properties of dental gypsum were studied with 23.00% water–powder ratio, 0.30% CPS (copolymer water reducer), 0.10% BR (borax cross-linker agent), and 2.00% Nano-SiO_2_ and 0.90% aluminum stearate as experimental control group.

**Figure 4:**
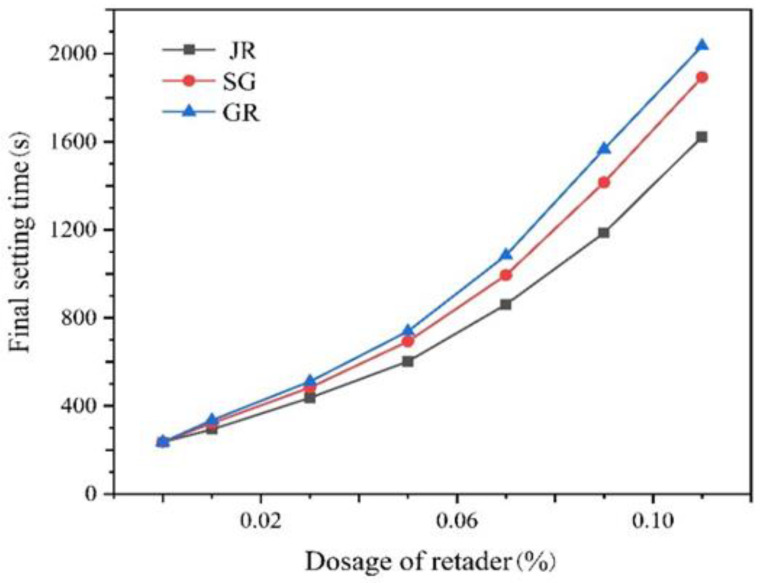
Effects of different kinds of retarders on the final setting time of dental gypsum at different dosages (ww^−1^).

Figure 4 shows that the retarding effect on dental gypsum of three kinds of retarders was significant when the dosage was at a ratio of 10 thousandths, belonging to a high-efficiency retarder. At the same dosage, the retardation effect of GR was the strongest, followed by SG and JR.

**Figure 5:**
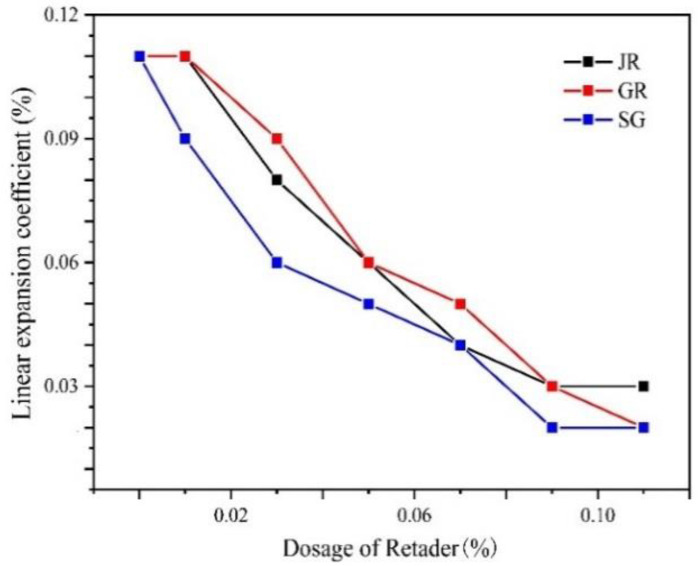
Effects of different kinds of retarders on the linear expansion coefficient of dental gypsum at different dosages (ww^−1^).

Figure 5 indicates that three retarders reduced the linear expansion coefficients of gypsum in varying degrees. When the dosage was more than 0.09%, the linear expansion coefficient tended to be 0.02–0.03%, which belonged to zero expansion. When the dosage was 0.09%, the setting time was about 20 min, which was not suitable for clinical use. Considering the strength loss and the actual cost of the solidified body, SG was selected as the best retarder. The final setting time was about 8 min when the dosage was 0.03%, and the expansion coefficient was 0.06%. According to the actual requirements of the final setting time range (6–10 min), the dosage shall not be higher than 0.05%.

### 2.5. Effect of Water Reducer on Linear Expansion Coefficient

It is well known that a water reducer is a substance that facilitates the kneading of powder and liquid to decrease the amount of water for kneading, thereby enhancing the strength of the composition after setting; however, its effect on the thermal expansion of gypsum has been rarely studied.

Concerning the experience of building gypsum, four representative water reducers were selected to study the modification effect of the linear expansion coefficient of dental gypsum. The control group was 23% water–powder ratio gypsum with 0.03% SG, 0.10% BR, 0.90% aluminum stearate, and 2.00% Nano-SiO_2._

**Figure 6:**
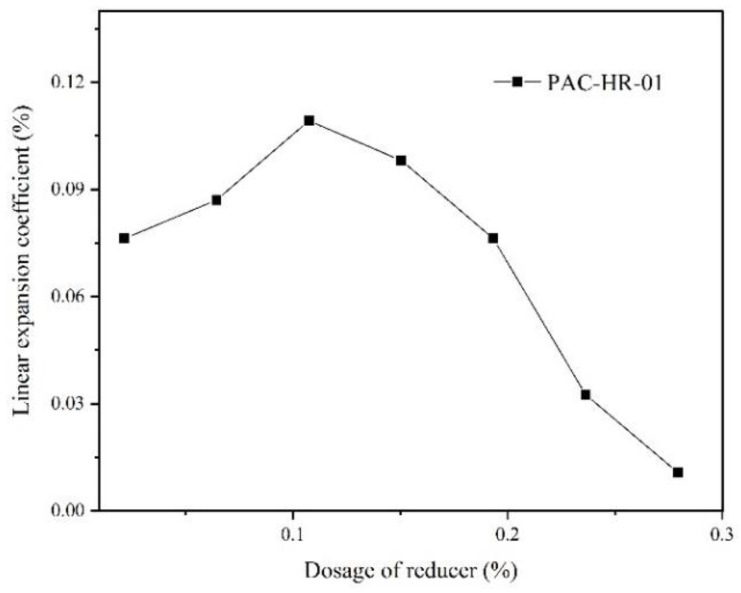
The effect of PAC-HR-01 on the linear expansion coefficient of dental gypsum at different dosages (ww^−1^).

Figure 6 shows that the expansion coefficient of gypsum increased first and then decreased after adding PAC-HR-01 (polycarboxylate water reducing agent). When the dosage reached 0.30%, the expansion coefficient was as low as 0.02%, belonging to zero expansion, which proved that the increase in dosage of PAC-HR-01 could effectively inhibit the formation of setting expansion. However, the slump flow at dosage of 0.30% was as high as 89 mm, and the slurry was easy to leak into the mold and increase the loss rate of the mold. Considering the influence of PAC-HR-01 on fluidity and setting expansion of dental gypsum, the dosage should not exceed 0.15%.

**Figure 7:**
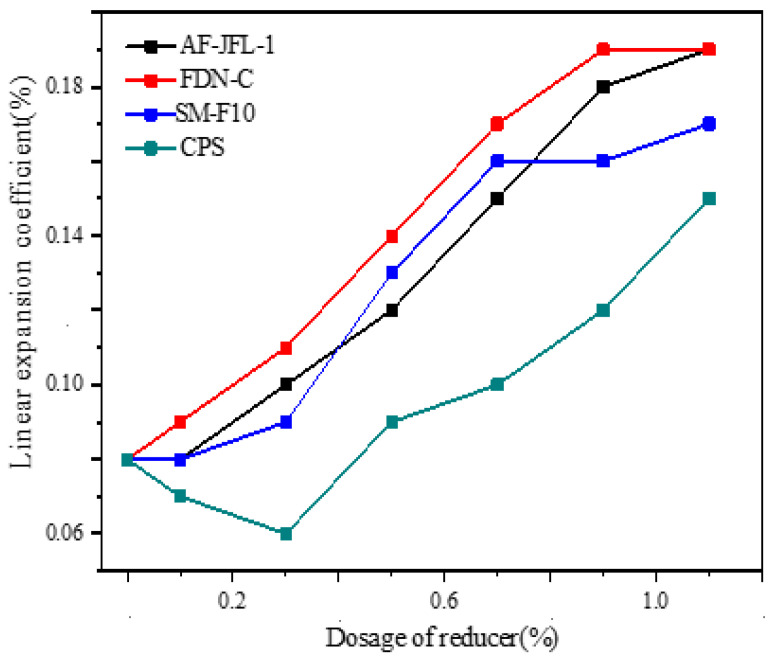
The effect of AF-JFL-1, FDN-C, SM-F10, and CPS on the linear expansion coefficient of dental gypsum at different dosages (ww^−1^).

It can be seen from Figure 7 that, with the increase in AF-JFL-1 (anthracene water reducing agent), FDN-C (naphthalene water reducing agent), and SM-F10 (sulfonated melamine water reducing agent) dosage, the linear expansion coefficient of dental gypsum increased continuously. It can also be seen that the influence of CPS (copolymer water reducer) on the linear expansion coefficient was optimum. The linear expansion coefficient was minimized at a dosage of 0.30% CPS. Meanwhile, CPS reduced the viscosity while improving the fluidity of the slurry, leading to minute independent bubbles being dispersed on the solidified material, thereby providing better kneading performance and operability.

Analysis of the reasons for the different trends in the two stages after adding CPS: When the dosage of CPS was less than 0.30%, the calcium sulfate hemihydrate was fully hydrated; the number of crystals of calcium sulfate dihydrate increased while the length-diameter ratio decreased, thereby optimizing the microstructure of the hardened body of the gypsum. Therefore, an appropriate amount of water reducer could reduce the expansion value of gypsum. With further increase in CPS, a large number of acidic molecules would be adsorbed on the crystal surface in the form of chemisorption, which reduced the free energy of the crystal surface, leading to coarsening of the crystal. Therefore, the linear expansion of gypsum became more obvious when the dosage of CPS exceeded 0.30%.

### 2.6. Effect of Reinforcer on the Gelation Characteristics of Dental Gypsum

Due to the high solubility of hydrated products in the hardened gypsum paste, the gypsum mold in the saturated water state would have a large degree of strength loss, and the introduction of accelerants and retarders would also cause a certain degree of strength loss. In order to reduce the solubility of the dental gypsum hydration product and to increase the bonding force between the hydration products, an appropriate amount of reinforcer should be added there to make up for this defect.

#### 2.6.1. Linear Expansion Coefficient

In this experiment, MCC (Microcrystalline cellulose), NCC (Nanocellulose), GA (Gum Arabic), and three inorganic nanomaterials were used to improve the strength of dental gypsum, and the expansion performance of the mold gypsum sample was accordingly improved. The control group was 23.00% water–powder ratio, 0.30% CPS, 0.03% SG, 0.10% BR, and 0.90% aluminum stearate.

**Figure 8:**
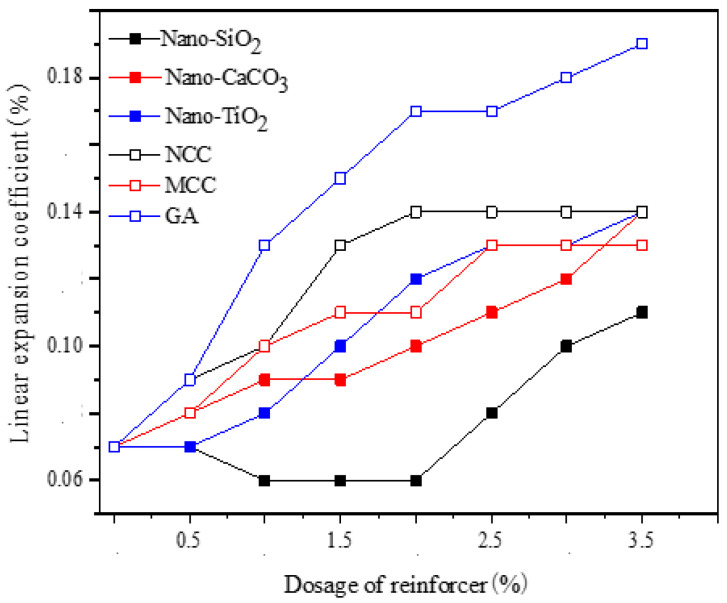
The effect of reinforcer on the linear expansion coefficient of α-hemihydrate gypsum at different dosages (ww^−1^).

Figure 8 indicates that the linear expansion coefficient of dental gypsum increased with Nano-CaCO_3_, Nano-TiO_2_, MCC, NCC, and GA. GA was the most conducive for the formation of linear expansion, and the expansion coefficient increased slowly when the dosage of GA exceeded 2.00%, followed by NCC. The effects of MCC, Nano-CaCO_3_, and Nano-TiO_2_ were similar. When the dosage of Nano-SiO_2_ was less than 2.00%, the linear expansion coefficient would not decrease, obviously, whereas the overall growth rate was the smallest.

#### 2.6.2. Mechanical Properties

**Figure 9:**
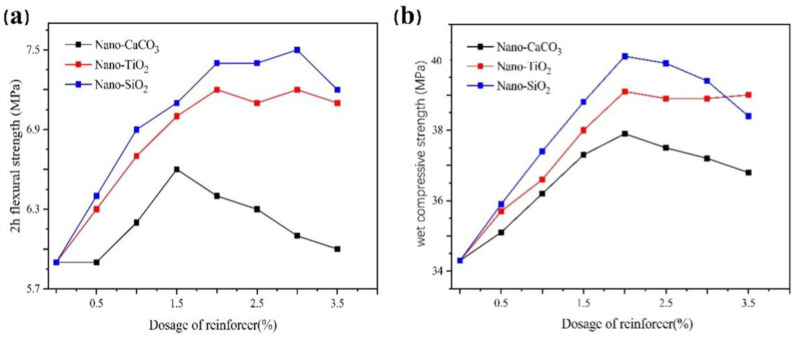
The effect of inorganic nano-materials on the mechanical strength of dental gypsum at different dosages (ww^−1^) (**a**) 2 h flexural strength, (**b**) wet compressive strength.

Figure 9 shows that the flexural strength and wet compressive strength of the dental gypsum increased first and then remained unchanged or decreased after adding inorganic nanomaterials. The enhancement effect of Nano-SiO_2_ was most obvious, followed by Nano-TiO_2_ and Nano-CaCO_3_.

When the dosage of Nano-SiO_2_ was 2.00%, the 2 h flexural strength of dental gypsum was 7.4 MPa, increasing by 25.40%. Moreover, the wet compressive strength was increased to the maximum, up to 40.1 MPa, by 16.90%. At this time, the slump flow of slurry was 67 mm, whose operating state has met clinical requirement. With the increase in Nano-SiO_2_, 2 h flexural strength increased slightly and then decreased slowly, and wet compressive strength decreased directly. This was because the fluidity of the slurry was damaged after the increase in dosage, and small holes appeared in the cross section of the molded specimen, resulting in a gradual decrease in mechanical strength.

**Figure 10:**
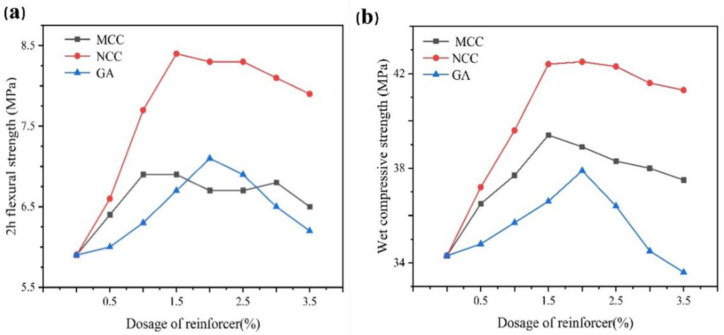
The effect of NCC, MCC, and GA on the mechanical strength of dental gypsum at different dosages (ww^−1^) (**a**) 2 h flexural strength, (**b**) wet compressive strength.

MCC, NCC, and GA as reinforcers were also used to regulate the mechanical properties of dental gypsum, and the influence results were shown in Figure 10.

The reinforcement effect of NCC was the best. With the increase in NCC, MCC, and GA, 2 h flexural strength and wet compressive strength of gypsum model increased first and then fluctuated or decreased. When the dosage of MCC was 1.50%, the mechanical properties of gypsum were optimum, and 2 h flexural strength and compressive strength were 6.9 MPa and 39.4 MPa, respectively. After adding 2.00% GA, 2 h flexural strength and wet compressive strength were 7.1 MPa and 37.9 MPa, respectively, which rose to the highest level. When the dosage of NCC was 1.50%, 2 h flexural strength increased to 8.4 MPa, which was 42.40% higher than the contrast sample, and 42.4 MPa in wet compression. In particular, the improved 2 h flexural strength was obviously much better than most of the commercial dental gypsum products.

**Figure 11:**
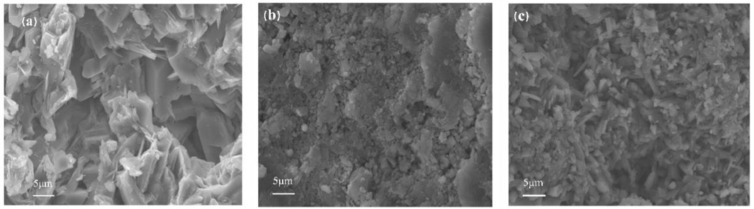
SEM images of gypsum (**a**) 0%, (**b**): 1% Nano-SiO_2_, (**c**): 1% NCC (multiple = 2.7 k, scale = 5.00 μm).

Figure 11 shows the micro morphology of the cross section of dental gypsum after hydration for 2 h with different reinforcement materials under the electron scanning microscope. Without reinforcer, the calcium sulfate dihydrate crystal was a lamellar and layered structure with loose crystal network and large pores between grains. When 1.00% Nano-SiO_2_ was added, the structure became dense, and internal defects were reduced. After adding 1.00% NCC, the array of crystal particles in the solidified body was more regular, uniform, and orderly; the crystal spacing was further reduced, and the structure was more complete, indicating that the reinforcement effect of NCC was more obvious, which was consistent with the experimental results.

## 3. Discussion

Before designing any composite structure, three interdependent factors must be considered: (1) a selection of the suitable matrix and dispersed materials; (2) a choice of appropriate fabrication and processing methods; and (3) both the internal and external structural design of the device itself [25,26]. As an essential index to evaluate the internal structure of dental gypsum, the linear expansion coefficient is a key element [27,28]. It determines whether a certain gypsum material can be used as a high-precision dental restoration material [29]. Taking the linear expansion coefficient of dental gypsum as an index, the water–powder ratio was 23.00%, and the dosage of BR was 0.10%. SG, CPS, Nano-SiO_2_, and aluminum stearate were selected as four influencing factors to carry out the orthogonal experiment of four factors and three levels. The factors and horizontal distribution results of the experimental scheme were shown in Table 1. Take the average value of the three experiments as the final result, accurate to 0.001%.

It can be seen from the range of R in Table 2 that, among the factors affecting the setting expansion, the addition amount of aluminum stearate was most significant, followed by CPS, SG, and Nano-SiO_2_. It can be concluded from the value of k that the best process solution for the low-expansion and high-strength dental gypsum was D3C3B2A3. It means that the optimum ingredient ratio for the formula sample is 0.90% aluminum stearate, 0.30% CPS, 0.03% SG, 2.00% Nano-SiO_2_, and 0.10% BR. The test was repeated three times, and the average linear expansion coefficient of the dental gypsum was 0.06%, which was within the error tolerance range.

To further prove the advantages of the selected scheme, the linear expansion of each sample was measured continuously for 72 h, and 8 time intervals were selected for analysis. We compared the blank control group (CK) with three kinds of dental gypsum products with good general performance in order to study the differences of setting expansion and their stability. These three dental gypsum products with good performance and the best commercial quality are Dentona, Heraeus, and Bowin. We purchased all three products, formulated them into gypsum materials with a 23% water–powder ratio, and tested their linear expansion coefficients. The results indicate that, among these three dental gypsum products, Dentona has the best performance, and its expansion value is only reduced to 0.13–0.20% when it meets the high compressive strength of 39.2 MPa. The linear expansion coefficients of Heraeus and Bowin are above 0.2% in 2 h, the size changes are greater, and the retardation time is longer.

It can be seen from Table 2 that the linear expansion coefficients of the blank control group, Heraeus, and Bowin were more than 0.20%, which were not suitable for the production of high-precision dental models [1]. The linear expansion value of Dentona increased from 0.13% to 0.20% within 72 h, which could not meet the occlusal requirements of precision dental castings for clinical applications because it was entirely higher than the 0.06% expansion value required by the application level. The linear expansion of formula sample was basically completed within 12 h, and its linear expansion coefficient is as low as only 0.07%. In the subsequent time frame, the formula sample performance is very stable, no expansion phenomenon occurs, and the linear expansion rate is only 16.70%, which can be used as a high-precision clinical dental gypsum material.

Therefore, the dental gypsum prepared in this study with low linear expansion coefficient, high strength, and stable performance, after being optimized by compound admixtures, was obviously superior to most representatives of commercial dental gypsum products.

## 4. Conclusions

The dental gypsum prepared in this study has the best comprehensive properties of low expansion and high strength. The linear expansion coefficient was reduced from 0.26% to 0.06%, inclined to zero expansion with a stable expansion property. Meanwhile, 2 h flexural strength increased from 6.7 to 7.4 MPa, resulting in compression resistance, increased to 40.1 MPa. It was suitable for the manufacture of high-precision dental prostheses. After using Nano-SiO_2_, the linear expansion coefficient may be slightly reduced, but 2 h flexural strength and wet compressive strength are increased significantly.

The dental gypsum material prepared in this study has excellent properties. It will help many patients have a more comfortable treatment experience if it can be applied in the dental field.

## 5. Materials and Methods

### 5.1. Materials

**Figure 12:**
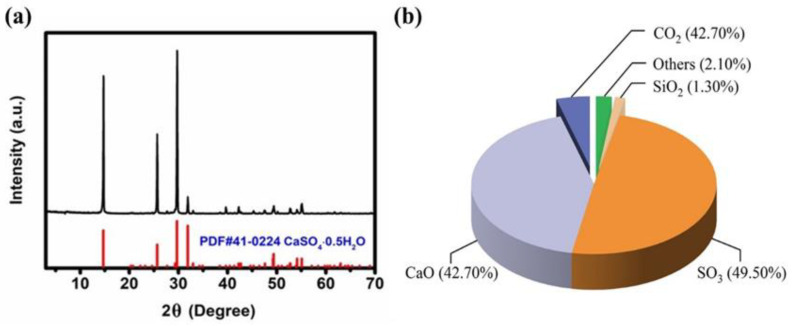
(**a**) XRD experimental results of α-dental gypsum (35 kV (30 mA)^−1^, 8° min^−1^, 0.02° step^−1^), (**b**) main chemical composition.

Dental gypsum was provided by Tangshan Xinghua Gypsum Co., Ltd. (Hebei, China), technical grade. The physical properties of the gypsum powder are shown in Table 3. The chemical composition and phase composition of dental gypsum were analyzed by X-ray fluorescence spectrometry (XRF) and X-ray diffraction (XRD), as shown in Figure 12.

According to the XRD test results, the main phase component in the sample was hemihydrate gypsum. It can be seen from the three strong peaks and the corresponding normalized intensity values that the crystallinity of dental gypsum sample was good.

Polycarboxylate water reducing agent (PAC-HR-01) was purchased from Nantong Runfeng Petrochemical Co., Ltd., Nantong, China, technical grade. Anthracene water reducing agent (AF-JFL-1) was purchased from Tianjin Feilong Concrete Admixture Co., Ltd., Tianjin, China, technical grade. Sulfonated melamine water reducing agent (SM-F10) was purchased from Shanghai Chenqi Chemical Co., Ltd., Shanghai, China, technical grade. Naphthalene water reducing agent (FDN-C) and Borax cross-linker agent (BR) were purchased from Yousuo Chemical Technology Co., Ltd., Beijing, China, technical grade. Multi-component copolymer water reducer (CPS) was purchased from Shijiazhuang Chenxiang Nonmetallic Mineral Research Institute.

Bone glue retarder (GR) was purchased from Suzhou Rongguang Chemical Co., Ltd., Suzhou, China, technical grade. Sodium gluconate retarder (SG) was purchased from Pudong Xingbang Chemical Development Co., Ltd., Shanghai, China, technical grade. Methionine retarder (JR) was purchased from Shanghai Yanyu New Building Materials Co., Ltd., Shanghai, China, technical grade.

Potassium sulfate and aluminum stearate were purchased from Sinopharm Chemical Reagent Beijing Co., Ltd., Beijing, China, analytical reagent; calcium sulfate dihydrate was purchased from Beihua Kaiyuan Chemical Co., Ltd., Beijing, China, analytical reagent.

Nano-TiO_2_, Nano-SiO_2_, and Nano-CaCO_3_ were purchased from Jinan Texing Chemical Co., Ltd., Jinan, China, technical grade. Nanocellulose (NCC) was provided by National Center for Nanoscience technology, analytical reagent. Microcrystalline cellulose (MCC) was purchased from Beijing Coupling Technology Co., Ltd., Beijing, China, analytical reagent. Gum Arabic (GA) was purchased from Tianjin Lichang Chemical Co., Tianjin, China, Ltd., technical grade.

Dentona, Heraeus, and Bowin dental gypsum products were purchased from Beijing Xin Kang Venture Trading Co., Ltd., Beijing, China, technical grade.

### 5.2. Instruments

Vicat apparatus, model SN09; high-strength gypsum deformer, model BX-100a. Both were provided by Shanghai Rongjida Instrument Technology Co., Ltd., Shanghai, China. Electric bending test machine was provided by Shanghai Shenrui Test Equipment Manufacturing Co., Ltd., Shanghai, China, model SD-75. Universal testing machine was provided by Shimadzu Co., Ltd., Beijing, China, model AGS-10KNG. The X-ray diffractometer (XRD) was provided by Rigaku Co., Ltd., Beijing, China, model D(max-rB). The X-ray fluorescence spectrum analyzer (XRF) was provided by Rigaku Co., Ltd., Beijing, China, model ZSX Primus II. The scanning electron microscope (SEM) was provided by JEOL Co., Ltd., Beijing, China, model JSM-IT300.

### 5.3. Methods

#### 5.3.1. Preparation of Low-Expansion Gypsum Powder for Dentistry

First, the water reducer, retarder, accelerant, and reinforcer were pulverized to a 100 mesh particle size in a tank pulverizer. The above additives were weighed according to a specific ratio, mixed with 50 g dental gypsum in the mixer for 60 s, and the mixer was then shut down for 5 min. An addition of 100 g α-dental gypsum powder was added to the mixer followed by 60 s of mixing, and the mixer was shut down for 5 min. A 300 g dental gypsum powder was again added to the mixer followed by 60 s of mixing and shut down for 5 min, then mixed up with 550 g of dental gypsum for 60 s, and shut down for 5 min once again; finally, the low-expansion and high-strength gypsum powder for dental model was prepared.

#### 5.3.2. Mixing and Molding

An amount of distilled water was added appropriately to the mixing bowl according to different water–powder ratio (23–27%), the low-expansion high-strength gypsum powder was then slowly added into the mixing bowl to avoid large air retention. The powder was first placed in water for 30–60 s to ensure that the solvent water, gypsum powder, and additives have a penetration and surface pre-wetting effect of preventing agglomeration. Through preliminary performance tests, we found that the difference in performance between the 23–27% water–powder ratio gypsum materials was small, so we chose to use the smallest water–powder ratio for the experiments in order to save cost and maximize clinical application. The mixture was manually stirred and a homogeneous mixture with a weak-gel state was obtained after 60–90 s. The prepared slurry was slowly poured into a Vicat apparatus, high-strength gypsum deformer, and triple mortar mold coated by mineral oil, subsequently removing the surface bubbles by shaking. After scraping off the overflow slurry with a spatula, cover the surface of the test piece with a layer of PTFE film and place it at room temperature (20 ± 5 °C) for airtight curing and start molding.

## Figures and Tables

**Table 1:** Orthogonal scheme factor and horizontal distribution table.

Run	A (SG) %	B (CPS) %	C (Nano-SiO_2_) %	D (C_54_H_105_AlO_6_) %	Expansion Value %
1	1 (0.01)	1 (0.2)	1 (1.0)	1 (0.3)	0.131
2	1 (0.01)	2 (0.3)	2 (1.5)	2 (0.6)	0.078
3	1 (0.01)	3 (0.4)	3 (2.0)	3 (0.9)	0.064
4	2 (0.02)	1 (0.2)	2 (1.5)	3 (0.9)	0.082
5	2 (0.02)	2 (0.3)	3 (2.0)	1 (0.3)	0.114
6	2 (0.02)	3 (0.4)	1 (1.0)	2(0.6)	0.102
7	3 (0.03)	1 (0.2)	3 (2.0)	2 (0.6)	0.076
8	3 (0.03)	2 (0.3)	1 (1.0)	3 (0.9)	0.058
9	3 (0.03)	3 (0.4)	2 (1.5)	1 (0.3)	0.125
*K*_1_	0.273	0.289	0.291	0.370	
*K*_2_	0.298	0.250	0.285	0.256	
*K*_3_	0.259	0.291	0.254	0.204	
*k*_1_	0.091	0.096	0.097	0.123	
*k*_2_	0.099	0.083	0.095	0.085	
*k*_3_	0.086	0.097	0.085	0.068	
Δ*R*	0.013	0.014	0.012	0.055	

**Table 2:** Linear expansion coefficient within 72 h (%).

Sample		CK	Heraeus	Dentona CAD/CAM	Bowin KKK	Formula Sample
Time interval (h)	2	0.26	0.26	0.13	0.22	0.06
	6	0.26	0.26	0.15	0.24	0.06
	12	0.27	0.27	0.15	0.24	0.07
	24	0.27	0.27	0.18	0.25	0.07
	36	0.29	0.28	0.18	0.26	0.07
	48	0.30	0.28	0.19	0.27	0.07
	60	0.31	0.28	0.20	0.27	0.07
	72	0.31	0.28	0.20	0.27	0.07
growth rate(%)		19.23	7.69	53.85	22.73	16.67

**Table 3:** Physical property of α-dental gypsum used in this paper.

Tested Parameters	Properties
Standard consistency water demand (%)	27
Initial setting time (s)	634
Final setting time (s)	930
Linear expansion coefficient (%)	0.26
2 h flexural strength (MPa)	6.70
Wet compressive strength (MPa)	36.30
